# Highly Efficient Elimination of As(V) and As(III) from Aqueous Media Utilizing Fe-Ti-Mn/Chitosan Composite Xerogel Beads

**DOI:** 10.3390/gels12020112

**Published:** 2026-01-27

**Authors:** Chunting Chen, Junbao Liu, Hongpeng Cao, Zhaojia Li, Jianbo Lu, Wei Zhang

**Affiliations:** School of Environmental and Material Engineering, Yantai University, Yantai 264005, China; 202400361060@s.ytu.edu.cn (C.C.); 202500361077@s.ytu.edu.cn (J.L.); 13561953760@s.ytu.edu.cn (H.C.); 202376502129@s.ytu.edu.cn (Z.L.)

**Keywords:** xerogel beads, composite metal oxides, chitosan, As(V), As(III), elimination

## Abstract

Inorganic arsenic species, As(V) and As(III), present significant toxicity and carcinogenic risks in water, making their effective removal critical for global water safety. This study introduces Fe-Ti-Mn/chitosan composite xerogel beads (FTMO/chitosan) designed to overcome the limitations of conventional single-component adsorbents, particularly for simultaneous removal of As(V) and As(III), and to address solid–liquid separation challenges common with powdered adsorbents. The xerogel beads feature a rough, porous surface composed of agglomerated nanoparticles. Batch tests demonstrated that the Freundlich model provided a better fit for the adsorption process, with max uptake capacities of 22.6 mg/g and 16.2 mg/g for As(III) and As(V) at 25 °C, respectively, outperforming most reported adsorbents. Adsorption kinetics were fast, reaching equilibrium within 24 h and fitting well with a pseudo-second-order kinetic model. The adsorption efficiency was strongly influenced by solution pH and the existence of minor coexisting anions. Mechanistically, As(V) removal occurred via inner-sphere surface complexation through the substitution of surface hydroxyl groups, whereas As(III) removal involved a coupled oxidation-adsorption process: MnO_2_ oxidized As(III) to As(V), which was then adsorbed onto the material surface. Furthermore, the adsorbent confirmed excellent regeneration capacity and operational stability, illuminating its promising potential for frequent utilization in water treatment and environmental remediation applications.

## 1. Introduction

Arsenic, a nonmetallic element, is widely and abundantly distributed in nature. It primarily enters aquatic environments through natural geochemical processes, including weathering, volcanic eruptions, microbial activity, and desorption, as well as through various anthropogenic activities, such as industrial effluents, agricultural pesticide application, and animal feed additives [1,2]. Currently, arsenic contamination, defined as concentrations exceeding 50 μg/L, has been documented in several countries and regions worldwide, including Chile, Argentina, Bangladesh, and China [3,4]. Aside from Bangladesh, China faces one of the most severe risks of arsenic contamination, stemming from both human activities and natural sources. The Qinba Mountain area in Shaanxi Province is among the earliest nationally recognized arseniasis-endemic regions. Here, the average arsenic concentration in stone coal mined in southern Shaanxi reaches 117.82 mg/kg (with a max of 297 mg/kg), significantly exceeding the global average arsenic content in coal. Due to insufficient industrial management and limited environmental protection awareness, groundwater arsenic contamination frequently occurs in this region, with documented cases of arsenic poisoning among local residents [5]. Prolonged consumption of arsenic-contaminated drinking water can lead to cancers of the skin, lung, kidney, and liver, as well as numerous non-carcinogenic health issues affecting the respiratory, endocrine, and reproductive systems. Furthermore, individuals living in high-risk areas may develop chronic arsenism through inhalation, dermal contact, food consumption, and other exposure routes [6]. To mitigate the health risks posed by arsenic pollution, the world health organization, China, the US environmental protection agency, the European Union, Japan, and other authorities have implemented stricter drinking water standards, lowering the max allowable arsenic concentration from 50 μg/L to 10 μg/L [7].

In the natural environment, arsenic primarily exists in two oxidation states: As(V) and As(III). The speciation of arsenic in aqueous environments is largely influenced by redox potential (Eh) and pH. Relative to negatively charged As(V), As(III), which exists as a neutral, uncharged molecular species, is approximately ten times more toxic, more soluble and mobile, and less likely to be adsorbed or immobilized by natural minerals such as iron, aluminum, and manganese oxides [8]. Currently, common arsenic removal methods in water treatment include coagulation-sedimentation, ion exchange, biological treatments, adsorption, and membrane separation [9]. The selection of an optimal removal technology depends on specific external conditions and water quality characteristics, and often involves combining multiple methods to address complex arsenic contamination. Among them, adsorption technology has advantages such as operational simplicity, regeneration’s ease, and cost-effectiveness. It is particularly suitable for small communities and dispersed rural households with limited space, making it widely used in mountainous and rural areas with decentralized water supplies [10]. Traditional adsorbents like activated carbon and alumina have been effective; however, increasingly stringent drinking water standards challenge these materials to consistently meet safety requirements. To reduce costs and enhance removal efficiency, a range of novel multifunctional arsenic adsorbents have been developed, including natural and synthetic iron-, titanium-, and manganese-based materials, as well as composite and polymer-based adsorbents.

The physicochemical properties of inorganic As(V) and As(III) in natural water vary significantly, posing challenges for treatment using single-component adsorbents, which often struggle to simultaneously remove multiple arsenic species due to intrinsic limitations [11]. To expand the applicability and effectiveness of adsorbent materials, an increasing number of novel metal oxide composites have been developed. The synergistic interactions among components in these composites can significantly enhance arsenic removal efficiency [12,13,14,15]. For instance, Cai et al. [12] successfully fabricated a nanostructured Fe-Cu-Mn composite oxide adsorbent, achieving max adsorption capacities of 115.2 mg/g and 158.5 mg/g for As(V) and As(III) at 25 °C under neutral conditions. Liu et al. [13] developed an iron-manganese composite oxide (FMO) capable of rapidly reducing arsenic concentrations from 1000 μg/L to below 10 μg/L within 60 min at 25 °C. Additionally, Mn-doped MgAl-layered double hydroxides demonstrated a high adsorption capacity for As(V) of 166.94 mg/g (T = 25 °C, adsorbent dosage = 0.1 g/L, pH = 7.0), following pseudo-second-order (PSO) kinetics [14]. A new Fe-Mn-Ce oxide-modified biochar composite was also synthesized, which exhibited a max adsorption capacity of 8.74 mg/g for As(III) (adsorbent dose = 5 g/L, contact temperature = 25 °C, pH = 7.0) [15]. However, despite these promising capacities, the practical application of these nanoadsorbents is limited due to the fact that they tend to agglomerate, are difficult to separate, and may be cytotoxic if they escape into the environment [16,17,18].

To overcome these physical limitations, powdered adsorbents are frequently immobilized onto porous solid supports that possess excellent hydraulic properties through coating, loading, or impregnation techniques [16,17,18,19,20]. Chitosan, a biodegradable aminopolysaccharide derived from the alkaline deacetylation of chitin, the most abundant natural biological polymer, can be easily formed into particles or thin films. It offers advantages such as high abundance, biodegradability, environmental friendliness, and alkali resistance, making it widely used for immobilizing powdered nanoparticles [16,17]. For instance, Verduzco-Navarro et al. [19] evaluated chitosan-magnetite hydrogel beads for arsenate removal from aqueous solutions, finding that adsorption followed the Langmuir isotherm with a max capacity of 66.9 mg As/g at 25 °C and initial pH of 7.0. Rawat et al. [18] developed iron alginate beads (IIAB) and chitosan-coated iron beads (IICB) for the removal of arsenic species. Langmuir modeling indicated that the adsorption capacities followed a superior order: IICB for As(V) > IICB for As(III) > IIAB for As(V) > IIAB for As(III) (adsorbent dosage = 1 g/L, initial pH = 6.7 ± 0.2), highlighting the enhanced performance of chitosan-coated beads, especially for As(V) (20.93 mg/g). Additionally, Song et al. [20] synthesized magnetic composite microspheres (MTHCNPs-2) combining TiO_2_, Fe_3_O_4_, and chitosan/quaternized chitosan derivatives, which achieved max adsorption capacities of 34.61 mg/g and 33.68 mg/g for As(V) and As(III) at 40 °C, respectively.

In our recent preliminary experiments, Fe-Ti-Mn/chitosan composite xerogel beads (FTMO/chitosan) were synthesized, and they demonstrated effective arsenic removal performance, accompanied by efficient solid–liquid separation. These results indicate that FTMO/chitosan possesses significant potential as a practical adsorbent for arsenic remediation in water treatment. However, there has been no prior report or systematic study on this material in the literature. Therefore, this study focuses on: (1) synthesizing FTMO/chitosan adsorbents through a two-step sequential process; (2) systematically evaluating their arsenic adsorption efficiency and operational performance (e.g., adsorption kinetics, adsorption isotherm, thermodynamics, influence of solution pH, impact of coexisting ions, regeneration test, and column experiment); and (3) elucidating the underlying removal mechanisms for both As(V) and As(III) using FTIR and XPS technology.

## 2. Results and Discussion

### 2.1. The Arsenic Elimination by FTMO/Chitosan Material with Series Mass Ratio

**Figure 1:**
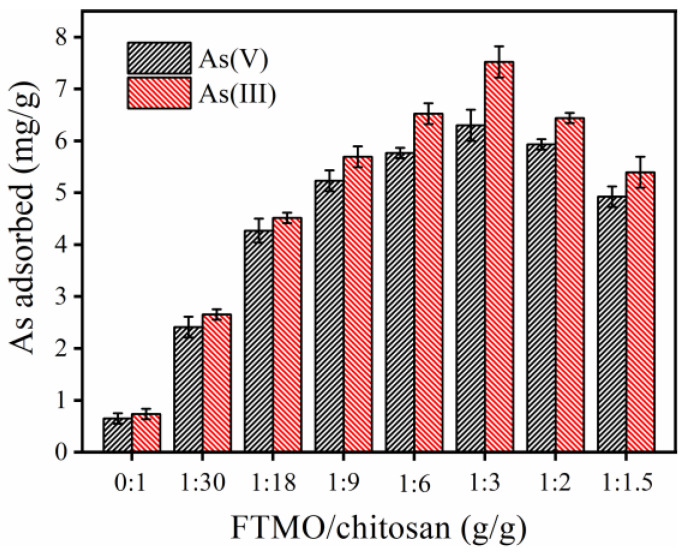
Effect of FTMO/chitosan mass ratio on As(V) and As(III) adsorption by the FTMO/chitosan adsorbent. Initial As concentration: about 10 mg/L; adsorbent dose: 1 g/L; pH: 7.0 ± 0.1; shaking speed: 170 rpm; temperature: 25 °C.

A series of FTMO/chitosan adsorbents with varying mass ratios were evaluated for their adsorption efficiencies toward As(V) and As(III), and the results are presented in Figure 1. Pure chitosan exhibited poor arsenic removal capacity, with adsorption amounts of merely 0.65 mg/g for As(V) and 0.75 mg/g for As(III). Incorporating FTMO significantly enhanced the removal performance. The adsorbent with an FTMO/chitosan mass ratio of approximately 1:3 demonstrated the max adsorption capacity for both arsenic species. Increasing the FTMO content beyond this ratio compromised material formation and potentially limited adsorption efficiency. Consequently, the FTMO/chitosan composite with a 1:3 mass ratio was selected for all subsequent experiments.

### 2.2. Characterization of FTMO/Chitosan Material

**Figure 2:**
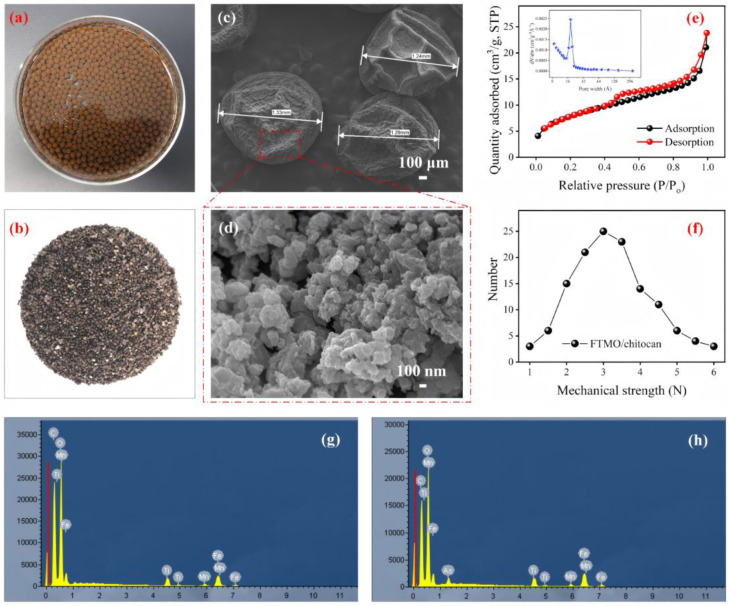
Photographs of the wet FTMO/chitosan (**a**) and the dry FTMO/chitosan (**b**), SEM micrograph ((**c**): FTMO/chitosan, (**d**): FTMO/chitosan localised magnification) of the FTMO/chitosan, N_2_ adsorption–desorption isotherms of the FTMO/chitosan (**e**), the mechanical strength distribution of the FTMO/chitosan (**f**) and EDS surface analysis ((**g**): without adsorption, (**h**): with As(III) adsorption) of the FTMO/chitosan.

SEM and EDS were employed to characterize the surface morphology and properties of the adsorbent. As depicted in Figure 2a–c, the FTMO/chitosan xerogel beads display a uniform granular morphology, with bead sizes spanning from 1 to 1.5 mm. This characteristic facilitates efficient solid–liquid separation. The surfaces of the beads are rough and porous, and they are characterized by the conspicuous agglomeration of numerous nanoparticles sized between 100–500 nm (Figure 2d). EDS analysis (Figure 2g,h) detected characteristic peaks for carbon (C), iron (Fe), titanium (Ti), manganese (Mn), and oxygen (O) both before and after arsenic adsorption, confirming these elements as the primary constituents of the composite material. This finding is in line with the result of the XPS analysis (Supplementary Figure S1). After arsenic adsorption, a distinct arsenic (As) peak appears, indicating effective uptake of arsenic onto the adsorbent surface. The FTMO/chitosan exhibited a BET specific surface area of 28.7 m^2^/g and a pore volume of 0.028 cm^3^/g. Based on the desorption average pore width calculated by the BET method (4V/A), the adsorbent had a pore size of 1.98 nm, which was larger than the diameter of arsenic species (Figure 2e). Additionally, mechanical strength testing of randomly selected xerogel beads revealed values between 2.2 and 3.5 N (Figure 2f), demonstrating high mechanical strength and structural stability sufficient to resist fragmentation during practical use.

### 2.3. The Inorganic Arsenic Removal Performance by the FTMO/Chitosan Material

#### 2.3.1. Adsorption Kinetics of Inorganic As(V) and As(III)

**Figure 3:**
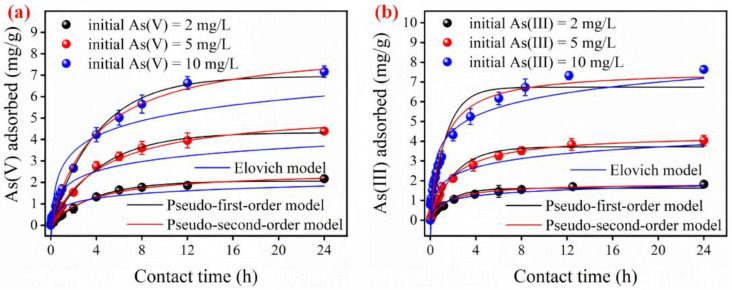
Kinetics of As(V) and As(III) removal on the FTMO/chitosan adsorbent. (**a**) As(V) fitted with pseudo-first-order, pseudo-second-order and Elovich models, and (**b**) As(III) fitted with pseudo-first-order, pseudo-second-order and Elovich models. Initial As concentration: about 2–10 mg/L; adsorbent dose: 1 g/L; pH: 7.0 ± 0.1; shaking speed: 170 rpm; temperature: 25 °C.

The batch adsorption kinetics of the FTMO/chitosan material for As(V) and As(III) were investigated, and the results are presented in Figure 3. The FTMO/chitosan adsorbent exhibited outstanding adsorption efficiency for both arsenic species. The adsorption process can be comprehensively divided into two distinct stages: a rapid adsorption phase followed by a slower adsorption phase. During the rapid phase, the adsorbent attains approximately 50% of its equilibrium adsorption capacity within the initial 4 h, which is especially remarkable for As(III) removal. In the subsequent slow phase, the adsorption rate gradually decreases as the system approaches equilibrium. Bessaha et al. [21] observed that the adsorption amount gradually increased with the increasing contact time. To comprehensively characterize the full adsorption behavior, a contact time of 24 h was employed for all experiments.

The classical pseudo-first-order, PSO, and Elovich models can elucidate the removal mechanisms and pinpoint the possible rate-controlling steps during the adsorption of As(V) and As(III) by the FTMO/chitosan adsorbent, for example, whether physical diffusion or chemical bonding plays a dominant role [22,23,24]. The fitting results and error analyses are shown in Table 1 and Supplementary Table S1. As shown, the PSO kinetic model provided a superior fit for both As(V) and As(III) adsorption, evidenced by higher regression coefficients and lower sums of squared residuals compared to the other models. The estimated (Q_ecal_) adsorbed quantities are consistent with the experimental (Q_eexp_) adsorption capacity. Additionally, the high F-value (>2000) and highly significant *p*-value (Prob > F < 0.00001) further confirm the excellent agreement between the PSO model and the experimental data. The PSO model assumes that the adsorption rate is primarily controlled by chemical adsorption having electron transfer or electron sharing between the adsorbate and the adsorbent [23]. Thus, the adsorption of As(V) and As(III) onto the FTMO/chitosan material is likely governed by a chemical adsorption mechanism. Similar chemical adsorption behaviors have been reported in related systems, including Fe-Cu composite oxides for phosphorus removal [25], Fe-Zr composite oxides for arsenic adsorption [26], and exchanged zeolite adsorbent for Cr(VI) removal from aqueous solutions [21].

#### 2.3.2. The Adsorption Isotherm and Thermodynamics of Inorganic As(V) and As(III)

**Figure 4:**
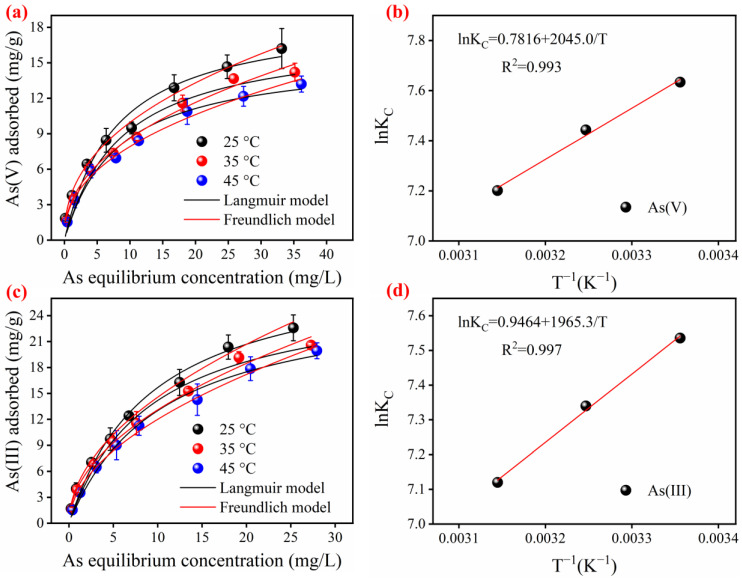
Adsorption isotherms of As(V) and As(III) by the FTMO/chitosan at three different temperatures. (**a**) As(V) fitted with Langmuir and Freundlich models, (**b**) As(V) thermodynamics curve, (**c**) As(III) fitted with Langmuir and Freundlich models, and (**d**) As(III) thermodynamics curve. Initial As concentrations: about 2–50 mg/L; adsorbent dose: 1 g/L; pH: 7.0 ± 0.1; shaking speed: 170 rpm; temperature: 25 °C, 35 °C and 45 °C.

The adsorption isotherms and thermodynamics of the FTMO/chitosan material for As(V) and As(III) are shown in Figure 4a–c. The material exhibits notably high adsorption capacities for both arsenic species, with a particularly strong affinity for As(III). To investigate the adsorption mechanisms of inorganic As(V) and As(III) on the adsorbent surface, the experimental isotherm data were fitted utilizing the widely accepted Freundlich and Langmuir models [27,28]. The fitting results and corresponding error analyses are summarized in Table 2 and Supplementary Table S2. The fitting findings show that the Freundlich model yields a higher regression coefficient and a smaller residual sum of squares compared to the Langmuir model, indicating a better fit for the adsorption isotherm data of both As(V) and As(III). Moreover, the Freundlich model fitting produces F-values exceeding 1000 and Prob > F values below 0.0001, further confirming its suitability for describing the adsorption behavior. Generally, the Freundlich model is appropriate for heterogeneous, multi-layer adsorption processes involving diverse adsorption sites and variable adsorption capacities on the adsorbent surface. Conversely, the Langmuir model undertakes monolayer adsorption on a homogeneous surface [27,28]. As illustrated in Figure 2d, the FTMO/chitosan material exhibits a rough, uneven surface composed of multiple components, consistent with the heterogeneous adsorption behavior observed. Accordingly, As(V) and As(III) are adsorbed via heterogeneous multi-layer mechanisms on this material. Comparable adsorption trends have been reported for the removal of As(V) and As(III) using Ce-Mn, Zr-Mn, and Ti-Mn composite adsorbents [29,30,31].

Figure 4 shows that the experimentally determined max direct adsorption capacities (Q_maxexp_) of the FTMO/chitosan material for As(V) and As(III) are 16.2 mg/g (at 25 °C) and 22.6 mg/g (at 25 °C), respectively. Earlier experimental outcomes (Figure 1) illustrated that pure chitosan has limited effectiveness in eradicating inorganic As(V) and As(III) from water, serving primarily as a structural support within the composite. The adsorption performance is mainly attributed to the FTMO component. Furthermore, compared to previously reported granular adsorbents for arsenic removal (summarized in Table 3), the FTMO/chitosan material exhibits superior adsorption capacity. These findings suggest that FTMO/chitosan is a promising candidate for practical arsenic removal applications.

It was also observed from Figure 4a–c that the adsorption capacities for both arsenic species decreased as the temperature increased. This macroscopically indicated that the arsenic adsorption process was exothermic in nature. Thermodynamic parameters, including the change in standard free energy (ΔG^θ^), enthalpy (ΔH^θ^), and entropy (ΔS^θ^), were determined and are presented in Figure 4b,d, as well as Supplementary Table S3. As shown, the negative values of ΔG^θ^ at all temperatures indicated the feasibility of the process and the spontaneous nature of both arsenic species adsorption onto FTMO/chitosan. The negative value of ΔH^θ^ implied that the adsorption processes of both As(III) and As(V) were exothermic. Moreover, the positive value of ΔS^θ^ suggested an increase in randomness at the solid/solution interface and reflected some internal structural changes in the adsorbent after arsenic adsorption [40].

#### 2.3.3. The Solution pH Influence on Inorganic Arsenic Elimination

**Figure 5:**
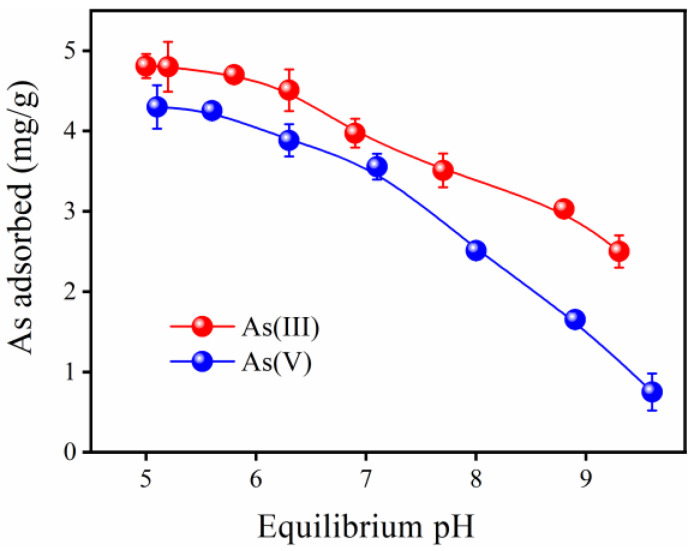
Effect of pH on As(V) and As(III) adsorption by FTMO/chitosan adsorbent. Initial As concentration: about 5 mg/L; adsorbent dose: 1 g/L; shaking speed: 170 rpm; temperature: 25 °C.

Figure 5 illustrates the impact of solution pH on the elimination of As(V) and As(III) by the FTMO/chitosan adsorbent. As shown, the adsorption efficiency for both As(V) and As(III) gradually decreases with increasing pH. The material exhibits excellent adsorption performance at lower pH values (5–7), but its capacity declines significantly at pH values above 7. Generally, the max adsorption of weak acid anions such as arsenite (As(III)) occurs near the p*K*_1_ value of the acid (p*K*_1_ = 9.2 for arsenite), indicating that metal oxides have the highest adsorption capacity for As(III) around this pH. In contrast, the adsorption capacity for strong acid anions like arsenate (As(V)) typically decreases as pH rises [41]. The similar removal trends for As(V) and As(III) suggest that the FTMO/chitosan adsorbent can effectively oxidize and adsorb As(III) in aqueous solutions. Within the tested pH range of 5 to 10, the dominant As(V) species are negatively charged H_2_AsO_4_^−^ and HAsO_4_^2−^ (arsenic acid p*K*_1_ = 2.2, p*K*_2_ = 6.98, and p*K*_3_ = 11.5) [42]. At lower pH, surface protonation of the adsorbent increases, enhancing positive surface charges and thereby strengthening electrostatic attraction to As(V) ions, resulting in high adsorption capacity. Conversely, at higher pH, surface deprotonation generates more negative charges, increasing electrostatic repulsion between the adsorbent and As(V), which reduces adsorption efficiency. Considering that natural water pH characteristically ranges from 6 to 8, the FTMO/chitosan adsorbent shows strong potential for practical arsenic removal applications.

#### 2.3.4. The Impact of Coexisting Ions on Inorganic Arsenic Elimination

**Figure 6:**
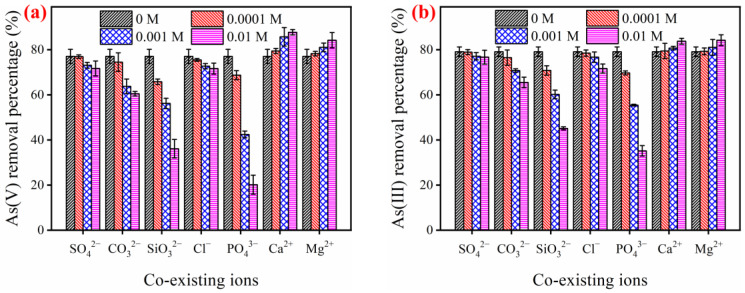
Effects of co-existing ions on As(V) (**a**) and As(III) (**b**) removal by FTMO/chitosan adsorbent. Initial As concentration: about 5 mg/L; adsorbent dose: 1 g/L; pH: 7.0 ± 0.1; shaking speed: 170 rpm; temperature: 25 °C.

Figure 6 illustrates the impact of common coexisting ions on the elimination of As(V) and As(III) by the FTMO/chitosan adsorbent. Regarding coexisting cations, both Ca^2+^ and Mg^2+^ enhance the elimination of As(V) and As(III), with the promotional effect becoming more pronounced as their concentrations increase. This enhancement is likely due to the adsorption of these cations onto the adsorbent surface, which increases surface electropositivity and strengthens the electrostatic attraction between the adsorbent and inorganic arsenic anions, thereby facilitating greater arsenic adsorption. In terms of coexisting anions, Cl^−^, SO_4_^2−^, and CO_3_^2−^ have minimal impact on the elimination of both As(V) and As(III). Even at concentrations up to 0.01 M, only slight inhibition is observed. However, PO_4_^3−^ and SiO_3_^2−^ ions significantly inhibit the removal of As(V) and As(III). For instance, when the concentrations of PO_4_^3−^ and SiO_3_^2−^ increase from 0 to 0.01 M, the elimination of As(V) by the FTMO/chitosan adsorbent decreases by approximately 73% and 53%, respectively. This inhibition can be elucidated by the chemical similarities among these ions: arsenic and phosphorus belong to the same principal group in the periodic table, while silicon and arsenic are diagonally adjacent in neighboring groups. Their corresponding ions, PO_4_^3−^, SiO_3_^2−^, and As(V), possess similar molecular structures and chemical properties [43]. As per previous research, the specific adsorption of anions onto metal oxides generally takes place via a ligand exchange mechanism involving surface hydroxyl groups [44]. Consequently, PO_4_^3−^ and SiO_3_^2−^ compete with As(V) for the identical adsorption sites (such as hydroxyl groups) on the adsorbent surface, thereby reducing arsenic uptake. Furthermore, the similar inhibitory trends observed for both As(V) and As(III) suggest that the neutral As(III) species are primarily oxidized to As(V) anions by the FTMO/chitosan material before being adsorbed.

#### 2.3.5. Regeneration Test

**Figure 7:**
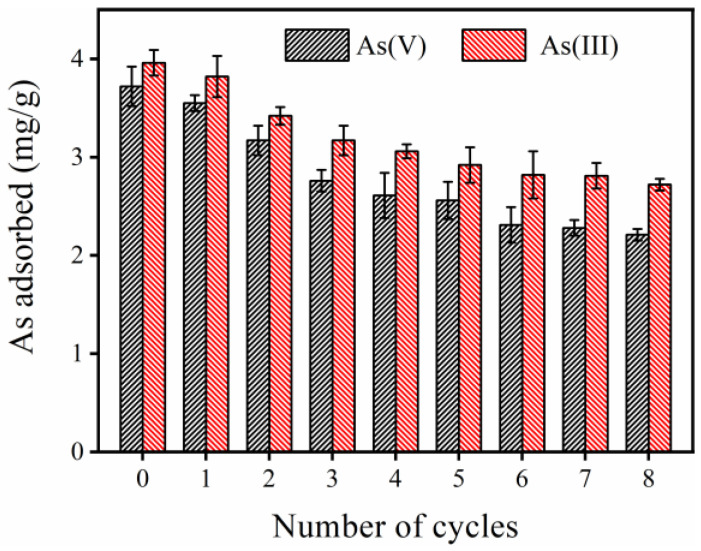
Adsorption capacity of As(V) and As(III) by the FTMO/chitosan adsorbent in eight successive adsorption–desorption cycles. Initial As concentration: about 5 mg/L; adsorbent dose: 1 g/L; pH: 7.0 ± 0.1; shaking speed: 170 rpm; temperature: 25 °C.

To evaluate the regeneration performance of the FTMO/chitosan adsorbent, consecutive adsorption–desorption experiments for As(V) and As(III) were carried out over eight cycles. As depicted in Figure 7, the initial adsorption capacities for As(V) and As(III) were 3.7 mg/g and 3.9 mg/g, respectively. Although the adsorption capacities gradually decreased as the number of cycles increased, the decrease was not significant. For instance, after the eighth cycle, the capacities still remained relatively high at 2.2 mg/g for As(V) and 2.7 mg/g for As(III), which indicates good regeneration stability. The observed reduction in adsorption capacity can be attributed to incomplete desorption: some arsenic ions remain bound to the adsorbent surface even after NaOH treatment, occupying active sites and thereby limiting the adsorption of new arsenic species in subsequent cycles. Notably, the FTMO/chitosan adsorbent maintained its original physical morphology and mechanical strength after multiple cycles. In addition to overcoming the solid–liquid separation challenges associated with powdered adsorbents, this material also overcomes common drawbacks of conventional supported adsorbents, such as poor alkali resistance and secondary pollution. These results demonstrate that the FTMO/chitosan adsorbent is reusable for arsenic removal in water treatment systems. Furthermore, the arsenic-rich solution recovered during desorption has the potential for further use in arsenic-related industrial applications.

### 2.4. Water Column Experiments

**Figure 8:**
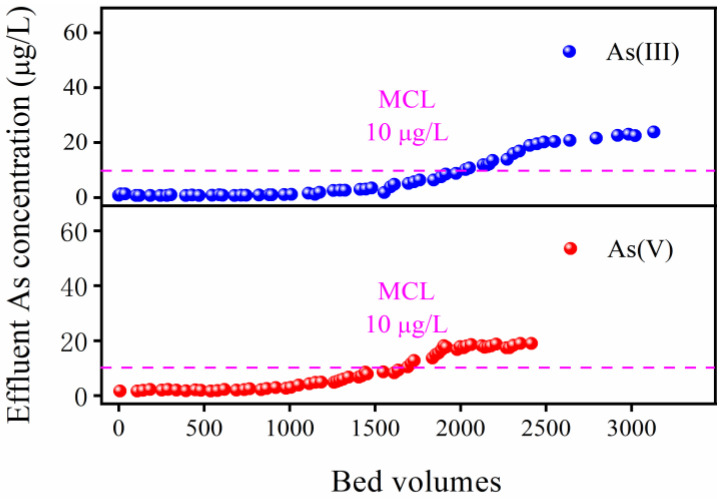
Breakthrough curves for arsenic adsorption by the FTMO/chitosan adsorbent from the simulated groundwater. Initial As concentration: about 220 μg/L; pH: 7.2 ± 0.1; retention time: 10 min; temperature: 25 °C.

Figure 8 represents the results of the column adsorption experiment evaluating the removal of As(V) and As(III) by the FTMO/chitosan adsorbent. The material exhibited excellent and sustained elimination for both inorganic arsenic species, maintaining stable purification performance over an extended period. Specifically, the concentration of As(III) in the effluent began to exceed the national drinking water standard of 10 μg/L after approximately 2000 column volumes (around 56 L), while As(V) concentrations surpassed the standard after about 1650 column volumes (roughly 46 L). Throughout the continuous operation exceeding 20 days, the FTMO/chitosan beads preserved their structural integrity with no signs of particle breakage, indicating robust mechanical strength and favorable physical properties. Additionally, post-treatment measurements showed that Mn and Fe concentrations in the treated water remained below national drinking water limits. These findings demonstrate the practical applicability and safety of the FTMO/chitosan adsorbent for effective arsenic removal in real-world water treatment settings.

### 2.5. Adsorption Mechanism

**Figure 9:**
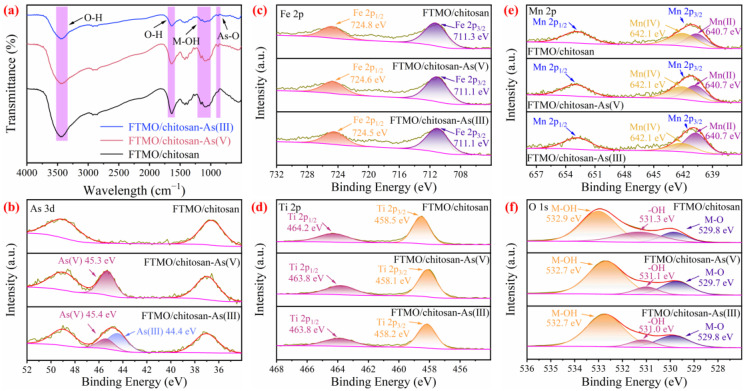
FTIR spectra (**a**) and XPS spectra of As 3d (**b**), Fe 2p (**c**), Ti 2p (**d**), Mn 2p (**e**), and O 1s (**f**) for FTMO/chitosan before and after adsorption of As(V) and As(III).

As depicted in Figure 9a, the broad peak at 3445 cm^−1^ can be attributed to adsorbed water and the sharp peak at 1638 cm^−1^ can be assigned to the deformation vibration of water molecules, indicating the presence of physisorbed water on the material. Additionally, three distinct absorption bands at 1160 cm^−1^, 1097 cm^−1^, and 1028 cm^−1^ are accredited to the bending vibrations of surface metal hydroxyl (M-OH) groups, where M represents Fe, Ti, or Mn [45]. Following the adsorption of As(V) and As(III), the intensity of these hydroxyl-related peaks diminishes gradually. Concurrently, a new absorption peak emerges at 874 cm^−1^, assigned to the bending vibration of As(V)-O bonds [46], indicating partial oxidation of As(III) to As(V). Furthermore, arsenate and arsenite ions coordinate with surface M-OH groups, forming M-O-As surface complexes. This coordination consumes surface hydroxyl groups, leading to attenuation or slight shifts in the O-H vibrational signals. Collectively, these observations confirm that the primary mechanism underlying arsenic removal by the FTMO/chitosan adsorbent is chemical coordination through surface hydroxyl groups.

The surface composition of the FTMO/chitosan adsorbent before and after adsorption of As(V) and As(III) was analyzed using XPS. The core-level spectra of As 3d, Fe 2p, Ti 2p, Mn 2p, and O 1s are presented in Figure 9. As shown in Figure 9b and detailed in Supplementary Table S4, a distinct As 3d peak appeared on the adsorbent surface following exposure to both As(V) and As(III), providing direct evidence of successful arsenic uptake. For As(III) adsorption, the As 3d binding energies (BEs) were measured at 45.4 eV and 44.4 eV, corresponding primarily to As(V) and As(III) species, respectively [47]. This observation strongly supports an oxidation-adsorption mechanism, whereby As(III) is oxidized to As(V) prior to adsorption, enabling the effective chemical removal of both arsenic species.

Figure 9c and Supplementary Table S5 present the Fe 2p spectra of the adsorbent before and after arsenic adsorption. In the pristine sample, the characteristic peaks at 724.8 eV (Fe 2p_1/2_) and 711.3 eV (Fe 2p_3/2_) confirm the existence of Fe(III) [48]. Following arsenic adsorption, a slight shift in the Fe 2p_3/2_ BE is observed, attributed to coordination between arsenic anions and surface Fe-OH groups, leading to the formation of Fe-O-As bonds. This electron redistribution, involving partial electron transfer from the more electronegative oxygen atoms to iron, highlights iron’s critical role as an adsorption site in the arsenic removal process.

The Ti 2p spectra, shown in Figure 9d and detailed in Supplementary Table S6, reveal that the Ti 2p_3/2_ and Ti 2p_1/2_ peaks of the pristine adsorbent are situated at 458.5 eV and 464.2 eV, respectively, consistent with the Ti(IV) oxidation state [49]. After arsenic adsorption, both peaks shift toward lower BEs by 0.3–0.4 eV, indicating an increased electron density around the Ti atoms. This shift suggests the formation of Ti-O-As surface complexes, confirming titanium’s active involvement in surface coordination during arsenic removal.

The Mn 2p spectra (Figure 9e and Supplementary Table S7) show that the pristine adsorbent has a Mn 2p_3/2_ BE at 641.0 eV, with a spin–orbit splitting, characteristic of Mn(II)/Mn(IV) oxides [50]. After adsorption of As(V), only negligible changes in the Mn 2p_3/2_ peak were observed, as As(V) remains fully oxidized and does not undergo redox reactions with Mn(IV). In contrast, following As(III) adsorption, significant changes occurred: the Mn(IV) signal intensity markedly decreased, while the Mn(II) signal increased, indicating the reduction of Mn(IV) to Mn(II). This suggests that Mn(IV) oxidizes As(III) to As(V), confirming an oxidation-adsorption synergistic mechanism. Thus, manganese plays a pivotal role in the oxidative transformation of As(III).

The O 1s spectrum (Figure 9f and Supplementary Table S8) reveals three overlapping components at BEs of 529.8 eV, 531.3 eV, and 532.9 eV, which correspond to metal-oxide (M-O), M-OH, and hydroxyl (-OH) groups, respectively [20,51]. Following arsenic adsorption, all O 1s peaks shifted toward lower BEs, indicating the active involvement of these oxygen-containing functional groups in arsenic binding, consistent with previously reported findings [52,53].

**Figure 10:**
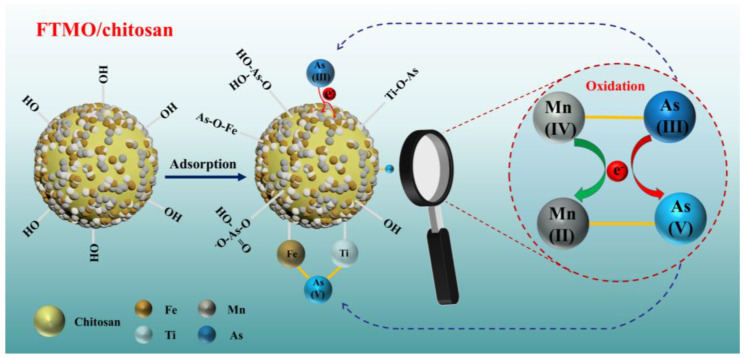
Schematic diagram of As(V) and As(III) removal mechanism by FTMO/chitosan adsorbent.

Based on this analysis and supported by earlier studies, the proposed mechanism for arsenic removal by FTMO/chitosan is illustrated in Figure 10. As(V) removal primarily occurs via ligand exchange, where arsenate ions replace surface hydroxyl groups, leading to the formation of inner-sphere surface complexes. For As(III), the removal involves an oxidation-adsorption process: Mn species in the material oxidize As(III) to As(V), which is subsequently adsorbed by the Fe and Ti components.

## 3. Conclusions

The FTMO/chitosan adsorbent was synthesized via a simple and environmentally friendly method, exhibiting uniform granularity and a porous, irregular surface composed of aggregated nanoparticles. It demonstrated rapid and efficient removal of As(V) and As(III) from aqueous solutions, achieving max adsorption capacities of 16.2 mg/g and 22.6 mg/g at 25 °C, outperforming most previously reported adsorbents. While PO_4_^3−^ and SiO_3_^2−^ ions significantly competed for adsorption sites, especially at higher concentrations, other common ions had minimal effects on arsenic uptake. The spent adsorbent was effectively regenerated using NaOH solution and successfully applied for arsenic removal in real water samples. FTIR and XPS analyses, together with batch adsorption results, revealed that As(V) removal occurs via ligand exchange with surface hydroxyl groups forming inner-sphere complexes, whereas As(III) removal involves an oxidation step followed by adsorption. This material exhibits remarkable performance under ideal conditions. Nevertheless, its long-term recyclability and structural stability in industrial applications still need further investigation. Future research ought to concentrate on optimizing the modification design of FTMO/chitosan composite materials, adopting cross-modification strategies to improve mechanical strength and adsorption capacity, and further exploring their incorporation into continuous water treatment systems to promote industrial application.

## 4. Materials and Methods

### 4.1. Materials and Chemicals

All chemicals utilized were analytical reagent grade and supplied by Sinopharm Chemical Reagent Co., Ltd. All chemical reagents used in the experiments are listed in Supplementary Table S9. Reagent solutions were made utilizing deionized water (18.25 MΩ·cm) sourced from a Milli-Q purification system (FLMO, Qingdao, China).

### 4.2. Preparation of FTMO/Chitosan Material

**Figure 11:**
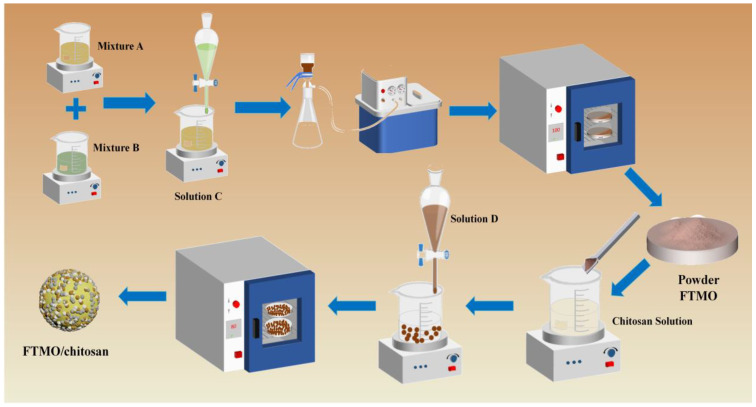
The preparation route of FTMO/chitosan adsorbent.

The FTMO/chitosan materials were synthesized via a two-step sequential process (Figure 11). Synthesis of FTMO powder: A solution (Mixture A) was prepared by dissolving FeCl_3_·6H_2_O, FeSO_4_·7H_2_O and Ti_2_(SO_4_)_3_ in water at a molar ratio of Fe:Ti:Mn = 4:2:1. Separately, KMnO_4_ and NaOH were dissolved in water to form Mixture B. While stirring rapidly, Mixture B was slowly added dropwise into Mixture A to yield Solution C. The combined solution was stirred for 30 min, and the pH was adjusted to 7.0. After standing for 1 h, the supernatant was decanted. The resulting solid precipitate was repeatedly washed with deionized water until no anions were detected in the washings. The solid was then separated, dried at 60 °C, ground, and sieved through an 80–100 mesh sieve to obtain the powdered FTMO material. Fabrication of FTMO/chitosan composite beads: Chitosan was dissolved uniformly in 0.2 M HCl (~2% *w*/*v*) by slow stirring. The powdered FTMO was then poured to the chitosan solution and stirred thoroughly to form Solution D. This mixture was gently dropped into 0.3 M NaOH solution, where wet FTMO/chitosan gel beads formed immediately. The beads were washed repeatedly with deionized water till neutral pH was reached and then dried at 60 °C to obtain the final FTMO/chitosan adsorbent. Additionally, a series of FTMO/chitosan materials with varying mass ratios were prepared following the same procedure.

### 4.3. Characterization of FTMO/Chitosan Material

Surface morphology of the FTMO/chitosan material was examined utilizing a field emission SEM (Nova NanoSEM 450, FEI, Waltham, MA, USA). Elemental composition and distribution were further analyzed by EDS with a data acquisition time of 20 min. The specific surface area, pore size, and pore volume were analyzed using an automatic physical adsorption apparatus (BET, Micromeritics ASAP 2460, Norcross, GA, USA). FTIR spectra of the FTMO/chitosan adsorbent before and after adsorption of As(V) and As(III) were recorded using a Nicolet IS10 spectrometer (Thermo Scientific, Waltham, MA, USA). XPS analysis was carried out to determine the valence states and surface elemental distribution using an ESCALAB 250Xi spectrometer (Thermo Fisher Scientific). The C 1s peak at 284.8 eV was utilized for internal energy calibration. Text S1 (Supplementary Material) elaborated on the operational details of the aforementioned techniques.

### 4.4. Batch Adsorption Experiments

All batch adsorption tests of arsenic by the FTMO/chitosan material were performed using deionized water (DI). Prior to use, 100 mL plastic bottles serving as containers were soaked in 3% nitric acid and thoroughly rinsed with DI to eliminate contaminants. Stock solutions of arsenic species (1 g/L) were prepared by dissolving the corresponding arsenic salts in DI and stored for up to one month. Unless otherwise noted, arsenic test solutions were prepared with 0.01 M NaNO_3_ as the background electrolyte. The pH of all solutions was maintained at 7.0 ± 0.1 by adjusting with NaOH or HNO_3_. Adsorbent dosage was fixed at 1 g/L for all experiments. Samples were incubated on a thermostatic shaker set at 25 °C and operated at 170 rpm for 24 h. After incubation, samples were filtered through 0.22 μm syringe membrane filters before concentration analysis. Adsorption isotherms and thermodynamics: Initial arsenic concentrations ranged from 2 to 50 mg/L. The operating temperature was set in the range of 25 °C to 45 °C. Effect of solution pH: Experiments were conducted across a controlled pH range of 5.0 ± 0.1 to 10.0 ± 0.1. Effect of coexisting ions: Concentrations of Ca^2+^, Mg^2+^, F^−^, Cl^−^, SO_4_^2−^, PO_4_^3−^, HCO_3_^−^ and SiO_3_^2−^ were varied from 0 to 0.01 M to assess their influence on arsenic adsorption. Adsorption kinetics: Solutions with arsenic concentrations of 2–10 mg/L were prepared in 2 L glass beakers, each containing 1 L of solution. Approximately 5 mL aliquots were collected at predetermined intervals over 24 h for analysis.

### 4.5. Reusability of FTMO/Chitosan Material

Arsenic solutions with a concentration of 5 mg/L were prepared in glass beakers, each containing 1.5 L of solution. The background electrolyte was 0.01 M NaNO_3_. The pH was adjusted and maintained at 7.0 ± 0.1 using NaOH and HNO_3_ solutions. A total of 1.5 g of the FTMO/chitosan adsorbent was poured to each solution. The mixtures were incubated in a thermostatic shaker at 170 rpm for 12 h. Following incubation, solid–liquid separation was conducted by filtering through a 0.22 μm cellulose acetate membrane. The arsenic concentration in the filtrate was then measured. For adsorbent regeneration, the used material was immersed in 200 mL of 0.5 M NaOH solution and stirred at 170 rpm for 6 h. After regeneration, the adsorbent was filtered, thoroughly rinsed with DI water till neutral pH was reached, and dried. The recycled adsorbent was subsequently reused for the next adsorption–desorption cycle.

### 4.6. Column Experiments

A total of 16 g of FTMO/chitosan material was packed into a customized plexiglass column measuring 20 cm in length with a total volume of 28 mL. Both ends of the column were securely plugged with soft porous gauze to prevent material loss while allowing fluid flow. Simulated groundwater containing arsenic, sourced from Shaanxi Province, was uniformly injected into the column using a peristaltic pump (BT100-1L-A, Longer Precision Pump, Halma, Buckinghamshire, UK). The flow rate was controlled to achieve an empty-bed contact time of 10 min per column volume (2.8 mL/min). Water samples were collected every 8 h for arsenic concentration analysis using appropriate instrumentation. The simulated groundwater had a pH of 7.2 and contained the following ions and concentrations: NO_3_^−^ (5 mg/L), HCO_3_^−^ (159 mg/L), HPO_4_^2−^ (0.13 mg/L), SiO_3_^2−^ (12 mg/L), Ca^2+^ (22 mg/L), Mg^2+^ (41 mg/L), Fe (0.3 mg/L) and Mn (0.015 mg/L). Arsenic concentration was adjusted to approximately 220 μg/L prior to injection.

### 4.7. Analytical Methods

Prior to arsenic analysis, the aqueous samples were carefully diluted to a max concentration of 200 μg/L and acidified with concentrated HNO_3_. Samples were then stored in acid-washed plastic vessels to prevent contamination. Total arsenic concentrations, including As (V) and (III), were measured utilizing ICP-MS (NexION 300X, PerkinElmer, Waltham, MA, USA). All analyses were completed within 24 h of sample collection to ensure data accuracy. The adsorption capacity and efficiency for arsenic were determined as follows:

(1) $$\mathrm{Adsorption}\;\mathrm{capacities}\;(q_{e})\;=\;\frac{\left(C_{0}\;-\;C_{t}\right)}{m}\;\times \;V$$

(2) $$\mathrm{Adsorption}\;\mathrm{efficiency}\;(\%)=\frac{\left(C_{0}-C_{e}\right)}{C_{0}}\;\times \;100$$

where C_0_, C_t_, and C_e_ (mg/L) represent the initial concentration of arsenic, the concentration at time t, and the equilibrium concentration, respectively; V (L) denotes the volume of the solution; and m (g) represents the mass of the adsorbent used.

## Figures and Tables

**Table 1:** Adsorption rate constant obtained from pseudo-first-order, pseudo-second-order and Elovich model for various initial concentrations of arsenic.

Initial Concentration (mg/L)	Q_eexp_ (mg/g)	Pseudo-First-Order			Pseudo-Second-Order			Elovich		
		k_1_ (h^−1^)	Q_ecal_ (mg/g)	R^2^	k_2_ (g/mg·h)	Q_ecal_ (mg/g)	R^2^	α (mg/(g·h))	β (mg/g)	R^2^
2 ^a^	2.17	0.26	2.07	0.9939	0.104	2.54	0.9960	4.17	3.15	0.8426
5 ^a^	4.39	0.23	4.33	0.9956	0.042	5.40	0.9984	7.74	1.53	0.8156
10 ^a^	7.16	0.24	6.95	0.9934	0.030	8.49	0.9977	13.81	0.96	0.8426
2 ^b^	1.82	0.59	1.62	0.9749	0.36	1.87	0.9940	5.72	3.58	0.9425
5 ^b^	4.04	0.47	3.72	0.9872	0.12	4.36	0.9979	10.49	1.56	0.9155
10 ^b^	7.63	0.73	6.74	0.9247	0.12	7.60	0.9611	38.35	0.94	0.9210

^a^ As(V), ^b^ As(III).

**Table 2:** Langmuir and Freundlich isotherm parameters for As(V) and As(III) adsorption onto FTMO/chitosan adsorbent.

Different Temperatures	Q_maxexp_ (mg/g)	Langmuir Model			Freundlich Model		
		Q_maxcal_ (mg/g)	K_L_ (L/mg)	R^2^	K_F_ ((mg/g)/(mg/L)^n^)	n	R^2^
25 °C ^a^	16.2	19.2	0.13	0.9572	3.8	0.42	0.9947
35 °C ^a^	14.2	17.7	0.11	0.9490	3.1	0.44	0.9812
45 °C ^a^	13.2	15.6	0.12	0.9718	3.0	0.42	0.9893
25 °C ^b^	22.6	30.4	0.11	0.9841	4.5	0.51	0.9953
35 °C ^b^	20.6	27.3	0.11	0.9837	4.2	0.50	0.9901
45 °C ^b^	19.9	26.7	0.10	0.9892	3.6	0.52	0.9931

^a^ As(V), ^b^ As(III).

**Table 3:** Comparison of maximum arsenic adsorption capacities for different adsorbents.

Adsorbent	Operating Conditions	Maximum Adsorption Capacity of As(III) (mg/g)	Maximum Adsorption Capacity of As(V) (mg/g)	Reference
FTMO/chitosan	C_0_: 2–50 (mg/L) T: 24 h K: 298.15 K	22.6 (pH 7.0)	16.2 (pH 7.0)	This study
PolyHIPE	C_0_: 0–4.5 (mg/L) T: 24 h K: indoor temperature	N/A	2.25 (pH 7.2)	[32]
Mag-FMBO	C_0_: 0–50 (mg/L) T: 24 h K: 323 K	16.03 (pH 7.0)	N/A	[33]
PVOH/Citric Acid	C_0_: 0–13 (mg/L) T: 24 h K: 298.15 K	N/A	14.1 (pH 7.0)	[34]
MPFS-PAA	C_0_: 0–0.1 (mg/L) T: 1 h K: indoor temperature	0.0925	N/A	[35]
MC-based MNPs	C_0_: 0–5 (mg/L) T: 3 h K: 298.15 K	N/A	3.2 (pH 5)	[36]
MS-HZO	C_0_: 0–10 (mg/L) T: 24 h K: 308.15 K	8.61 (pH 6)	6.97 (pH 6)	[37]
CS-Ti	C_0_: 0–50 (mg/L) T: 10 h K: 308.15 K	N/A	14.4 (pH 8)	[38]
Granular schwertmannite	C_0_: 0–25 (mg/L) T: 24 h K: 298.15 K	N/A	4 (pH 7.0)	[39]

N/A: not available.

## Data Availability

Data will be made available on request.

## Supplementary Materials

The following supporting information can be downloaded at: https://www.mdpi.com/article/10.3390/gels12020112/s1. Text S1: Operational details of characterization techniques; Figure S1: XPS spectra of FTMO/chitosan before and after As(V) and As(III) adsorption; Table S1: Analysis of kinetics error deviation data and estimation related to the adsorption of arsenic by FTMO/chitosan adsorbent using error functions and statistical functions; Table S2: Analysis of isotherms error deviation data and estimation related to the adsorption of arsenic by FTMO/chitosan adsorbent using error functions and statistical functions; Table S3: Thermodynamic constants for As(V) and As(III) adsorption onto FTMO/chitosan adsorbent; Table S4: As 3d peak parameters for the FTMO/chitosan before and after As(V) and As(III) adsorption; Table S5: Fe 2p peak parameters for the FTMO/chitosan before and after As(V) and As(III) adsorption; Table S6: Ti 2p peak parameters for the FTMO/chitosan before and after As(V) and As(III) adsorption; Table S7: Mn 2p peak parameters for the FTMO/chitosan before and after As(V) and As(III) adsorption; Table S8: O 1s peak parameters for the FTMO/chitosan before and after As(V) and As(III) adsorption; Table S9: Chemical reagents used in the experiments.
