# Supplementary material for: Highly Efficient Elimination of As(V) and As(III) from Aqueous Media Utilizing Fe-Ti-Mn/Chitosan Composite Xerogel Beads
Source: Gels. 2026 Jan 27;12(2):112. doi: 10.3390/gels12020112 (PMC12941213; doi:10.3390/gels12020112)
Supplement: Supplementary file 1 [file gels-12-00112-s001.zip › gels-4103207-supplementary.pdf]

Supplementary Material

Article

Highly Efficient Elimination of As(V) and As(III) from Aqueous Media Utilizing Fe-Ti-Mn/Chitosan Composite Xerogel Beads

Chunting Chen, Junbao Liu, Hongpeng Cao, Zhaojia Li, Jianbo Lu * and Wei Zhang *

School of Environmental and Material Engineering, Yantai University, Yantai 264005, China; 202400361060@s.ytu.edu.cn (C.C.); 202500361077@s.ytu.edu.cn (J.L.); 13561953760@s.ytu.edu.cn (H.C.); 202376502129@s.ytu.edu.cn (Z.L.)

* Correspondence: jianbolu@ytu.edu.cn (J.L.); weizhang@ytu.edu.cn or wzhang2020@126.com (W.Z.)

Contents

Text S1. Operational details of characterization techniques.

Figure S1. XPS spectra of FTMO/chitosan before and after As(V) and As(III) adsorption.

Table S1. Analysis of kinetics error deviation data and estimation related to the adsorption of arsenic by FTMO/chitosan adsorbent using error functions and statistical functions.

Table S2. Analysis of isotherms error deviation data and estimation related to the adsorption of arsenic by FTMO/chitosan adsorbent using error functions and statistical functions.

Table S3. Thermodynamic constants for As(V) and As(III) adsorption onto FTMO/chitosan adsorbent.

Table S4. As 3d peak parameters for the FTMO/chitosan before and after As(V) and As(III) adsorption.

Table S5. Fe 2p peak parameters for the FTMO/chitosan before and after As(V) and As(III) adsorption.

Table S6. Ti 2p peak parameters for the FTMO/chitosan before and after As(V) and As(III) adsorption.

Table S7. Mn 2p peak parameters for the FTMO/chitosan before and after As(V) and As(III) adsorption.

Table S8. O 1s peak parameters for the FTMO/chitosan before and after As(V) and As(III) adsorption.

Table S9. Chemical reagents used in the experiments.

Text S1. Operational details of characterization techniques.

SEM: Using a Schottky field emission filament, in high vacuum mode, the resolution can reach 1.0 nm at an acceleration voltage of 15 kV, 1.4 nm at an acceleration voltage of 1 kV, and in low vacuum mode, it can reach 1.5 to 1.8 nm at 30 kV. The accelerating voltage ranges from 50 eV to 30 kV, and the magnification factor is between 35 and 1,000,000 times.

BET (ASAP 2460): The sample to be tested is dried. An appropriate amount of the adsorption material is placed in the sample bottle, followed by degassing pre-treatment at room temperature. Subsequently, it is tested and analyzed under liquid nitrogen at low temperature. The degassing temperature ranges from room temperature to 400°C; the specific surface area analysis range is from 0.01 m^2/g to an upper limit; the pore size analysis range is from 3.5 Å to 5000 Å; the minimum detectable value of pore volume is 0.0001 cc/g.

FTIR: The sample to be tested is thoroughly dried. An appropriate amount of adsorption material and dry KBr powder (with a mass ratio of approximately 1:80) are ground and uniformly mixed. Subsequently, they are placed in a mold and pressed into a transparent thin sheet under a pressure of about 10×10^7 Pa. After that, the sample sheet is placed in the sample holder, and the test analysis is conducted on the machine. The spectral scanning range is from 4000 to 400 cm^{-1}, with a resolution of 4 cm^{-1}, and the scanning frequency is set between 16 and 32 times.

XPS: X-ray photoelectron spectroscopy (XPS) data were acquired using an ESCALAB 250Xi spectrometer with a monochromatic Al Kα X-ray source (1486.6 eV). The C1s binding energy of graphitic carbon at 284.8 eV was employed as a reference for calibration. Wide-scan spectra were recorded over an energy range of 0-1100 eV, with a pass energy of 80 eV and a step size of 1 eV. The high-resolution scans were conducted according to the peak being examined with pass energy 40 eV and step size 0.05 eV. XPS data process and peak fitting were carried out using a nonlinear least-squares curve-fitting program (XPSPEAK41 Software). Selected samples (i.e., initial arsenic concentrations of approximately 20 mg/L, adsorbent dose of 1 g/L, pH of 7.0 ± 0.1, and shaking speed of 170 rpm) were dried for further analysis.

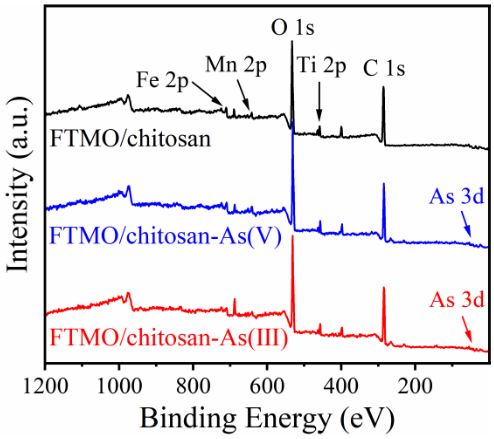

Figure S1. XPS spectra of FTMO/chitosan before and after As(V) and As(III) adsorption.

Table S1. Analysis of kinetics error deviation data and estimation related to the adsorption of arsenic by FTMO/chitosan adsorbent using error functions and statistical functions.

Modela/Initial Concentration (mg/L)		Adj. R-Square	Residual Sum of Squares	DF	Reduced Chi-Sqr	F Value	Prob > F
Pseudo-first-order Model[a]	2	0.9939	0.0560	18	0.00311	2760	4.1E-23
	5	0.9956	0.1770	18	0.00983	3583	3.9E-24
	10	0.9934	0.6573	18	0.03651	2555	8.1E-23
Pseudo-second-order Model[a]	2	0.9960	0.0371	18	0.00206	4173	9.9E-25
	5	0.9984	0.0639	18	0.00355	9933	4.1E-28
	10	0.9977	0.2340	18	0.01300	7192	7.4E-27
Elovich Model[a]	2	0.8426	1.3938	18	0.07743	102	1.5E-10
	5	0.8156	7.1003	18	0.39446	81	1.0E-9
	10	0.8426	15.1114	18	0.83952	103	1.5E-10
Pseudo-first-order Model[b]	2	0.9749	0.1672	18	0.00929	835	1.8E-18
	5	0.9872	0.4506	18	0.02503	1474	1.1E-20
	10	0.9247	7.5500	18	0.41945	340	5.0E-15
Pseudo-second-order Model[b]	2	0.9940	0.0397	18	0.00220	3548	4.2E-24
	5	0.9979	0.0757	18	0.00420	8823	1.2E-27
	10	0.9611	3.8982	18	0.21657	667	1.3E-17
Elovich Model[b]	2	0.9425	0.3570	18	0.01984	386	1.6E-15
	5	0.9155	2.8031	18	0.15573	229	1.6E-13
	10	0.9210	7.1628	18	0.39794	359	3.1E-15

[a]As(V), [b]As(III)

Table S2. Analysis of isotherms error deviation data and estimation related to the adsorption of arsenic by FTMO/chitosan adsorbent using error functions and statistical functions.

Modela	Different Temperatures	Adj. R-Square	Residual Sum of Squares	DF	Reduced Chi-Sqr	F Value	Prob > F
Langmuir Model[a]	25°C	0.9572	6.7573	6	1.12621	380	4.8E-7
	35°C	0.9490	6.4686	6	1.07811	319	8.1E-7
	45°C	0.9718	2.9359	6	0.48932	618	1.1E-7
Freundlich Model[a]	25°C	0.9947	0.8321	6	0.13869	3111	8.9E-10
	35°C	0.9812	2.3864	6	0.39774	871	4.0E-8
	45°C	0.9893	1.1149	6	0.18582	1631	6.2E-9
Langmuir Model[b]	25°C	0.9841	5.4583	6	0.90971	825	4.8E-8
	35°C	0.9837	4.7114	6	0.78523	831	4.6E-8
	45°C	0.9892	2.8327	6	0.47212	1253	1.4E-8
Freundlich Model[b]	25°C	0.9953	1.6100	6	0.26833	2804	1.2E-9
	35°C	0.9901	2.8457	6	0.47429	1379	1.0E-8
	45°C	0.9931	1.8158	6	0.30264	1956	3.6E-9

[a]As(V), [b]As(III)

Table S3. Thermodynamic constants for As(V) and As(III) adsorption onto FTMO/chitosan adsorbent.

As species	T (K)	ΔG^{θ} (kJ/mol)	ΔH^{θ} (kJ/mol)	ΔS^{θ} (J/(mol·k))
As(V)	298	-18.9	-17.0	6.50
	308	-19.0		
	318	-19.1		
As(III)	298	-18.7	-16.7	6.82
	308	-18.8		
	318	-18.8		

Table S4. As 3d peak parameters for the FTMO/chitosan before and after As(V) and As(III) adsorption.

Sample	Peak	B. E. (eV)	Percent (%)
**FTMO/chitosan**	As(V)	-	-
	As(III)	-	-
**FTMO/chitosan-As(V)**	As(V)	45.3	100
	As(III)	-	-
**FTMO/chitosan-As(III)**	As(V)	45.4	32.8
	As(III)	44.4	67.2

Table S5. Fe 2p peak parameters for the FTMO/chitosan before and after As(V) and As(III) adsorption.

Sample	Peak	B. E. (eV)
**FTMO/chitosan**	Fe $2p_{1/2}$	724.8
	Fe $2p_{3/2}$	711.3
**FTMO/chitosan-As(V)**	Fe $2p_{1/2}$	724.6
	Fe $2p_{3/2}$	711.1
**FTMO/chitosan-As(III)**	Fe $2p_{1/2}$	724.5
	Fe $2p_{3/2}$	711.1

Table S6. Ti 2p peak parameters for the FTMO/chitosan before and after As(V) and As(III) adsorption.

Sample	Peak	B. E. (eV)
**FTMO/chitosan**	Ti $2p_{1/2}$	464.2
	Ti $2p_{3/2}$	458.5
**FTMO/chitosan-As(V)**	Ti $2p_{1/2}$	463.8
	Ti $2p_{3/2}$	458.1
**FTMO/chitosan-As(III)**	Ti $2p_{1/2}$	463.8
	Ti $2p_{3/2}$	458.2

Table S7. Mn 2p peak parameters for the FTMO/chitosan before and after As(V) and As(III) adsorption.

Sample	Peak	B. E. (eV)	Percent (%)
**FTMO/chitosan**	Mn(II)	640.7	43.1
	Mn(IV)	642.1	56.9
**FTMO/chitosan-As(V)**	Mn(II)	640.7	44.8
	Mn(IV)	642.1	55.2
**FTMO/chitosan-As(III)**	Mn(II)	640.7	65.6
	Mn(IV)	642.1	34.4

Table S8. O 1s peak parameters for the FTMO/chitosan before and after As(V) and As(III) adsorption.

Sample	Peak	B. E. (eV)
	M-OH	532.9
**FTMO/chitosan**	-OH	531.3
	M-O	529.8
	M-OH	532.7
**FTMO/chitosan-As(V)**	-OH	531.0
	M-O	529.7
	M-OH	532.7
**FTMO/chitosan-As(III)**	-OH	531.1
	M-O	529.8

Table S9. Chemical reagents used in the experiments.

Reagent	Source	Identifier
**Sodium arsenate dibasic heptahydrate ($Na_2HAsO_4 \cdot 7H_2O$)**	Sigma Aldrich	CAS: 10048-95-0
**Sodium arsenite ($NaAsO_2$)**	Sigma Aldrich	CAS: 7784-46-5
**Iron(III) chloride hexahydrate ($FeCl_3 \cdot 6H_2O$)**	Sinopharm Chemical Reagent Co., Ltd.	CAS: 10025-77-1
**Iron(II) sulfate heptahydrate ($FeSO_4 \cdot 7H_2O$)**	Sinopharm Chemical Reagent Co., Ltd.	CAS: 7782-63-0
**Titanium sesquisulfate ($Ti_2(SO_4)_3$)**	Sinopharm Chemical Reagent Co., Ltd.	CAS: 13693-11-3
**Potassium permanganate ($KMnO_4$)**	Sinopharm Chemical Reagent Co., Ltd.	CAS: 7722-64-7
**Sodium hydroxide (NaOH)**	Sinopharm Chemical Reagent Co., Ltd.	CAS: 1310-73-2
**Hydrochloric acid (HCl)**	Sinopharm Chemical Reagent Co., Ltd.	CAS: 7647-01-0
**Nitric acid (HNO_3)**	Sinopharm Chemical Reagent Co., Ltd.	CAS: 7697-37-2
**Sodium nitrate ($NaNO_3$)**	Sinopharm Chemical Reagent Co., Ltd.	CAS: 7631-99-4
**Calcium nitrate tetrahydrate ($Ca(NO_3)_2 \cdot 4H_2O$)**	Sinopharm Chemical Reagent Co., Ltd.	CAS: 13477-34-4
**Magnesium nitrate hexahydrate ($Mg(NO_3)_2 \cdot 6H_2O$)**	Sinopharm Chemical Reagent Co., Ltd.	CAS: 13446-18-9
**Sodium chloride (NaCl)**	Sinopharm Chemical Reagent Co., Ltd.	CAS: 7647-14-5
**Sodium metasilicate nonahydrate ($Na_2SiO_3 \cdot 9H_2O$)**	Sinopharm Chemical Reagent Co., Ltd.	CAS: 13517-24-3
**Sodium sulfate (Na_2SO_4)**	Sinopharm Chemical Reagent Co., Ltd.	CAS: 7757-82-6
**Sodium hydrogen carbonate ($NaHCO_3$)**	Sinopharm Chemical Reagent Co., Ltd.	CAS: 144-55-8
**Disodium hydrogen phosphate (Na_2HPO_4)**	Sinopharm Chemical Reagent Co., Ltd.	CAS: 7558-79-4
**Potassium bromide (KBr)**	Sinopharm Chemical Reagent Co., Ltd.	CAS: 7758-02-3
